# Using Population Data to Reduce Disparities in Colorectal Cancer Screening, Arkansas, 2006

**DOI:** 10.5888/pcd9.110256

**Published:** 2012-08-16

**Authors:** Paul Greene, Paulette Mehta, Karen Hye-cheon Kim Yeary, Zoran Bursac, Jianjun Zhang, Geoff Goldsmith, Ronda Henry-Tillman

**Affiliations:** Author Affiliations: Paulette Mehta, Central Arkansas Veterans Health Care System, Little Rock, Arkansas; Karen Hye-cheon Kim Yeary, Zoran Bursac, Fay W. Boozman College of Public Health, University of Arkansas for Medical Sciences, Little Rock, Arkansas; Jianjun Zhang, Indiana University School of Medicine, Indianapolis, Indiana; Geoff Goldsmith, Ronda Henry-Tillman, University of Arkansas for Medical Sciences College of Medicine, Little Rock, Arkansas.

## Abstract

**Introduction:**

Colorectal cancer is a common disease, and incidence and death rates are higher in medically underserved populations. The colorectal cancer death rate in Arkansas exceeds the national rate. The objective of this study was to examine population characteristics relevant to the design and implementation of a state-sponsored colorectal cancer screening program that is responsive to medically underserved populations.

**Methods:**

Trained interviewers in 2006 conducted a random-digit–dialed telephone survey comprising items selected from the Health Information National Trends Survey to characterize demographic factors, health care variables, and colorectal screening history in a sample (n = 2,021) representative of the Arkansas population. Univariate and multivariate analyses identified associations among population characteristics and screening status.

**Results:**

Participants who were aged 50 to 64, who did not have health insurance, or who had an annual household income of $15,000 or less were significantly less likely than their counterparts to be in compliance with screening guidelines. Those who reported having a health care provider, having 5 or more health care visits during the past year, and receiving physician advice for colorectal screening were more likely to be in compliance with screening guidelines. Although a larger percentage of white participants were in compliance with screening guidelines, blacks had higher screening rates than whites when we controlled for screening advice.

**Conclusion:**

Survey results informed efforts to decrease disparities in colorectal cancer screening in Arkansas. Efforts should focus on reimbursing providers and patients for screening costs, encouraging the use of physicians as a point of entry to screening programs, and promoting a balanced approach (ie, multiple options) to screening recommendations. Our methods established a model for developing screening programs for medically underserved populations.

## Introduction

Colorectal cancer is a common disease, and incidence and death rates are higher in medically underserved populations (1). Early detection and removal of precancerous lesions can prevent the development of colorectal cancer, and regular screening can detect a malignant tumor at an early stage when it is more curable (2). For average-risk adults aged 50 or older, the fecal occult blood test (FOBT) is recommended annually, flexible sigmoidoscopy every 5 years, and colonoscopy every 10 years (3). Only 50% of the general population is routinely screened, and many people have never been screened (4,5). Screening rates are lower in populations that have limited access to health services, inadequate health insurance, low levels of formal education, and a high proportion of racial/ethnic minorities (6). Despite evidence that regular screening reduces colorectal cancer death rates, data to inform the development of population-based screening programs for medically underserved populations are limited (7,8).

The colorectal cancer incidence rate in Arkansas is higher than the national rate (49.8 per 100,000 vs 48.8 per 100,000) and so is the colorectal cancer death rate (18.9 per 100,000 vs 17.6 per 100,000). County colorectal cancer death rates range from 8.6 to 40.7 per 100,000 (9). To address these disparities, the University of Arkansas for Medical Sciences (UAMS), the Arkansas Department of Health, and the Arkansas State Legislature engaged in a collaborative effort to promote screening in populations at increased risk for colorectal cancer: racial/ethnic minority status, limited formal education, high levels of unemployment, low income, and rural residence. These populations are concentrated in 3 of the 5 Arkansas public health regions where colorectal screening rates are lowest (10). The objective of this study was to identify associations among demographic, health care, and screening characteristics of the Arkansas population to develop a state-sponsored screening program that increases screening rates among vulnerable populations.

## Methods

### Survey procedures

Trained interviewers used a computer-assisted telephone interview system to conduct a random-digit–dialed telephone survey in this cross-sectional observational study of the 5 public health regions in Arkansas in early 2006. The survey was administered to examine a broad range of issues related to colorectal cancer. Survey findings on geographic distribution of screening, knowledge of screening guidelines, and cancer-related attitudes are described elsewhere (10).

The survey comprised items selected from the Health Information National Trends Survey (11). Telephone exchanges (ie, landline telecommunications centers providing service in defined geographic areas) were stratified to ensure that each of the 5 public health regions was represented. Exchanges representing areas in which 15% or more of the population was black were oversampled to allow analysis of racial disparities.

Random-digit–dialed calls identified 4,592 private residences in which 1 or more people aged 50 or older lived. At 68% (3,128/4,592) of these residences, interviewers identified 1 age-eligible respondent, who was then invited to participate in the study. Interviewers completed surveys with 67% (2,092/3,128) of the respondents who were invited to participate. We excluded the following participants from analysis: people who reported a history of colorectal cancer (n = 40) and people who self-identified as multiracial (n = 4) or Hispanic (n = 27); the latter 2 groups were excluded because of their small sample size.

### Measures

Survey items assessed demographic and health care characteristics associated with colorectal screening (6,12-15).Demographic variables were age, sex, race, education level, employment status, annual household income, marital status, and health insurance status. We dichotomized the following variables: age (50-64 or ≥65), sex (male or female), race (white or black), education (<high school degree or ≥high school degree), employment (employed or unemployed), annual household income (≤$15,000 or >$15,000), marital status (married or has a partner or other), and health insurance (yes or no). The categories were established to determine which characteristics were most relevant to a state-sponsored colorectal cancer screening program. For example, participants aged 50 to 64 are not Medicare-eligible and may not be screened because of an inability to pay; participants who are unemployed and have no insurance also are likely to face financial barriers to screening; and participants who have an annual income of $15,000 or less meet federal poverty guidelines and are eligible for screening at no cost.

Health care variables addressed factors that may influence physician advice for colorectal screening and patient response to advice. Participants were asked whether they had a health care provider they saw most often, and they were asked to report the number of health care visits in the past year. The quality of the patient–provider interactions was assessed by asking questions on how often the health care provider listened carefully, explained things in an understandable way, showed respect, spent enough time, and involved patients in health care decisions. Response options for these 5 items were “never,” “sometimes,” “usually,” and “always.” In addition, participants could answer “don’t know,” or they could refuse to answer. We dichotomized these variables. For example, the 2 categories for number of health care visits in the past year were fewer than 5 visits and 5 visits or more. The vulnerable population was defined as people who had fewer than 5 visits because they have limited opportunities for screening advice. The 2 categories for each question on patient–provider interactions were “never” and “sometimes, usually, or always.” Participants who report that health providers never meet expectations experience a less supportive health care environment than those whose expectations were sometimes, usually, or always met by providers.

Screening advice was assessed by asking participants whether they had been advised by a physician to use a home FOBT kit, have a flexible sigmoidoscopy, or have a colonoscopy. We created a composite measure (“any advice”) for whether participants had been advised to use any of the 3 methods.

Screening status was assessed by asking participants whether they ever had been screened by FOBT, sigmoidoscopy, or colonoscopy. Participants who had been screened were asked to report when they were last screened. The survey identified current FOBT as screening in the past year and current sigmoidoscopy or colonoscopy as screening in the past 10 years. We created a composite measure for whether participants had ever been screened using any method and a composite measure for currently in compliance with guidelines using any method.

### Data analysis

We performed all analyses using SAS version 9.2 (SAS Institute Inc, Cary, North Carolina). Descriptive statistics provided weighted estimates of the proportion of the Arkansas population defined as vulnerable. Univariate and multivariate analyses examined the association of screening status with predictor variables (advice and race) and with covariates (demographics, use of health care services, and patient–provider interactions). Significance was set at .05 for all statistical tests.

Univariate analyses (Rao-Scott χ^2^ test) examined the association of each screening status variable with each screening advice variable and with demographic and health care characteristics. The association of predictor variables (advice and race) and covariates with screening status was comparable across the various measures of screening status. Therefore, we present only results on the association of predictor variables and covariates with current screening as a representative and clinically relevant measure of screening status. The associations of screening status variables with their respective screening advice variables also were comparable. Therefore, we used only the composite measure (“any advice”) as a measure of screening advice in subsequent multivariate analyses.

Multivariate logistic regression (Model 1) examined the association of current screening with predictor variables (“any advice” and race) and with covariates (demographic and health care characteristics). We conducted models 2 through 5 sequentially to determine which health care variables confounded the association of race with colorectal screening previously demonstrated in univariate analysis. 

## Results

### Sample characteristics

A larger percentage of white participants than black participants reported having a health care provider (Table 1). A larger percentage of blacks than whites reported their health care provider never takes enough time with them and never involves them in health care decisions. A smaller percentage of blacks than whites reported receiving provider advice on sigmoidoscopy, colonoscopy, or any screen, but no difference was found between blacks and whites for advice on FOBT.

**Table 1:** Demographic and Health Care Characteristics of Participants in Survey on Colorectal Cancer Screening Status, Arkansas, 2006^a^

Characteristic	Total, % (n = 2,021)	Black, % (n = 388)	White, % (n = 1,633)	*P* Value^b^
Aged 50–64 y	56.5	66.4	54.9	<.001
Male	35.8	28.9	36.9	.006
White	81.0	NA	NA	NA
Black	19.0	NA	NA	NA
<High school degree	16.5	34.4	13.1	<.001
Unemployed	19.4	25.3	18.5	<.001
Annual household income ≤$15,000	16.1	29.6	14.0	<.001
Married or has a partner	54.2	35.7	57.2	<.001
Insured	90.9	84.6	92.0	<.001
**Use of health care services**				
Has a health care provider	83.6	78.7	84.4	.01
Had ≥5 provider visits in past year	35.5	40.4	34.7	.05
**Patient–provider interaction^c^**				
Provider listens	98.5	97.1	98.8	.08
Provider explains	98.7	97.6	98.9	.10
Provider respects	98.9	98.1	99.0	.20
Provider takes time	97.4	95.5	97.7	.03
Provider involves	97.6	93.8	98.2	<.001
**Advice on screening**				
Fecal occult blood test	18.6	17.6	18.8	.63
Sigmoidoscopy	19.0	15.0	19.8	.05
Colonoscopy	51.3	36.8	53.7	<.001
Any advice	61.1	50.0	63.1	<.001
**Screening status**				
Current fecal occult blood test	14.8	16.0	14.7	.013
Current sigmoidoscopy or colonoscopy	45.1	35.4	46.7	<.001
Current on any screen	51.4	44.1	52.6	.006

Abbreviation: NA, not applicable.

^a^ Demographic and health care variables are dichotomized to examine vulnerable population segments relevant to a state-sponsored screening program. See Table 2 for each dichotomized pair.

^b^ Rao-Scott χ^2^ tests examined the association of race with demographic and health care variables.

^c^ Percentages of survey participants who answered “sometimes,” “usually,” or “always” vs “never.”

### Univariate analysis

The following demographic groups were significantly less likely than their counterparts to be in compliance with screening guidelines: those aged 50 to 64, women, blacks, singles, those who did not have a high school degree or have health insurance, and those who had an annual household income of $15,000 or less (Table 2).

**Table 2:** Screening Rates, by Demographic and Health Care Characteristic, Survey on Colorectal Screening Status (n = 2,021), Arkansas, 2006^a^

Characteristic	Received Screening, %	*P* Value^b^
**Age, y**		
50–64	48.7	.02
≥65	54.4	
**Sex**		
Male	56.2	.003
Female	48.8	
**Race**		
White	52.6	.006
Black	44.1	
**Education level**		
<High school degree	43.0	.001
≥High school degree	53.1	
**Employment status**		
Employed	52.6	.06
Unemployed	47.0	
**Annual household income, $**		
≤15,000	44.8	.008
>15,000	53.9	
**Marital status**		
Married or has a partner	55.1	<.001
Other	47.0	
**Has health insurance**		
Yes	54.0	<.001
No	27.3	
**Has a health care provider**		
Yes	55.7	.001
No	30.1	
**No. of health care visits in past year**		
≥5	60.2	.001
<5	46.6	
**Health care provider listens carefully**		
Never	39.3	.14
Sometimes, usually, or always	54.8	
**Health care provider explains things in an understandable way**		
Never	32.0	.04
Sometimes, usually, or always	55.0	
**Health care provider shows respect**		
Never	39.5	.20
Sometimes, usually, or always	54.7	
**Health care provider spends enough time**		
Never	47.1	.31
Sometimes, usually, or always	54.9	
**Health care provider involves patient in health care decisions**		
Never	44.6	.21
Sometimes, usually, or always	55.1	
**Received FOBT advice**		
Yes	87.7	<.001
No	43.2	
**Received sigmoidoscopy advice**		
Yes	80.0	<.001
No	44.8	
**Received colonoscopy advice**		
Yes	84.6	<.001
No	16.8	
**Received any advice**		
Yes	82.8	<.001
No	2.2	

Abbreviation: FOBT, fecal occult blood test.

^a^ Of the 2,021 survey participants, 1,009 were screened. Screening was defined as currently in compliance with guidelines using any method.

^b^ Rao-Scott χ^2^ tests examined differences in screening status associated with demographic and health care variables.

Many health care characteristics were associated with screening status. Participants who reported having a health care provider were more likely to be in compliance with screening guidelines than their counterparts as were participants who reported 5 or more health care visits during the past year. Participants who indicated that their health care provider gave understandable explanations sometimes, usually, or always were more likely to be in compliance with guidelines than those who indicated that their provider never gave understandable explanations. Participants who reported they received physician advice for colorectal screening were more likely to be in compliance with screening guidelines than participants who did not report receiving advice.

### Multivariate analysis

Among demographic measures, only male sex remained a significant predictor of screening status in Model 1 (Table 3). All health care characteristics and screening advice variables remained significant predictors of screening status in Model 1. Model 5 shows that whites were less likely to be in compliance with screening guidelines than blacks.

**Table 3:** Demographic and Health Care Characteristics Associated With Screening Status, Survey on Colorectal Screening Status (n = 2,021), Arkansas, 2006^a^

Characteristic	Model 1^b^ (n = 1,421)	Model 2^c^ (n = 1,604)	Model 3^c^ (n = 1,591)	Model 4^c^ (n = 1,434)	Model 5^c^ (n = 1,604)
**Demographic**					
White	0.60 (0.35–1.03)	1.08 (0.81–1.45)	1.15 (0.85–1.54)	1.19 (0.88–1.62)	0.59 (0.36–0.96)
Male	1.55 (1.03–2.34)	1.39 (1.10–1.75)	1.31 (1.04–1.66)	1.41 (1.10–1.81)	1.68 (1.14–2.48)
Aged ≥65 y	1.46 (0.98–2.17)	1.34 (1.07–1.69)	1.49 (1.18–1.88)	1.33 (1.05–1.79)	1.73 (1.20–2.51)
Not married or does not have partner	0.88 (0.57–1.35)	0.86 (0.68–1.09)	0.80 (0.63–1.02)	0.85 (0.66–1.09)	0.80 (0.54–1.19)
≥High school degree	0.99 (0.53–1.87)	1.28 (0.93–1.76)	1.44 (1.04–1.98)	1.29 (0.92–1.81)	0.93 (0.54–1.60)
Unemployed	1.05 (0.62–1.77)	0.89 (0.67–1.19)	0.96 (0.72–1.28)	0.91 (0.67–1.22)	0.87 (0.54–1.43)
Annual income ≤$15,000	0.64 (0.35–1.18)	0.74 (0.53–1.03)	0.73 (0.53–1.01)	0.74 (0.52–1.04)	0.65 (0.37–1.12)
**Health care**					
Has a health care provider	2.28 (1.23–4.22)	2.98 (2.18–4.09)	—	—	—
≥5 Visits with health care provider in past year	1.71 (1.13–2.56)	—	2.00 (1.58–2.53)	—	—
Health care provider explains things in an understandable way	6.06 (1.64–22.38)	—	—	2.47 (0.81–7.52)	—
Received any screening advice	352.3 (156.8–791.7)	—	—	—	257.6 (135.5–489.6)

^a^ All values are odds ratios (95% confidence intervals). All variables are dichotomous; see Table 2 for each dichotomous pair.

^b^ Model 1 simultaneously controls for demographic and health care variables.

^c^ Models 2 through 5 were sequentially conducted to identify health care variables that confound the association of race with colorectal screening.

## Discussion

Survey data collected from the 5 Arkansas public health regions expand the scientific literature on population-based colorectal cancer screening programs and provided a foundation for investigators and stakeholders to develop recommendations for a state-sponsored screening program. The study makes 3 novel contributions. One, we used categorical variables to examine vulnerable population segments relevant to the design and implementation of a state-sponsored colorectal screening program. Two, the higher screening rate found among blacks compared with whites when we controlled for screening advice provides new insight on the importance of promoting physician advice within a screening program intended to reduce disparities. Three, our process established a heuristic model for integrating state-specific data with general evidence-based guidelines to engage local stakeholders in efforts to inform a screening program. 

Our study resulted in 3 recommendations for a screening program that is responsive to medically underserved populations in Arkansas and consistent with guidelines published by the Centers for Disease Control and Prevention (16). One, establish state legislation that addresses reimbursement for colorectal cancer screening. Lower screening rates among unemployed and low-income participants suggest that cost may be a barrier to screening. Higher screening rates among Medicare-eligible participants relative to their counterparts aged 50 to 64 suggest that financial incentives for health care professionals and a reduction in out-of-pocket expenses for patients would increase screening rates. These findings are consistent with national data (4,6,8,17) and suggest that state-sponsored reimbursement may enhance population screening rates. Grass roots, health policy, and legislative stakeholders used state-specific data to establish the Arkansas Colorectal Cancer Prevention, Early Detection, and Treatment Act of 2009, which defines screening methods for a state-sponsored program. Survey data also were used by a community-based coalition to secure legislative appropriations to fund a pilot program that screens approximately 165 community residents per year.

Two, use primary care providers as a point of entry in population-based screening efforts. Consistent with studies reporting a strong association between physician recommendations and colorectal cancer screening (12,13), our study showed that health provider advice predicted various measures of screening status. Physician advice can be an effective point of entry for population-based screening efforts. Our data, which showed that 83.6% of participants had a health care provider and 35.5% had 5 or more health care visits in the previous year, support the feasibility of recommendations to integrate colorectal screening programs into existing clinical structures (7). Although a smaller proportion of blacks than whites in our study had a health care provider, 78.7% had a primary care provider and 40.4% had 5 or more health care visits in the previous year. That black participants were more likely than white participants to be in compliance with screening guidelines when controlling for physician advice differs from previous findings (4-6). Physician advice during routine health care visits can serve as an effective point of entry to a screening program for this population. The Arkansas pilot screening program reimburses specialists who provide endoscopic screening and primary care providers who identify screening-eligible patients, provide screening recommendations, and either initiate FOBT or refer patients for endoscopic screening.

Three, promote a balanced approach to screening recommendations. Because many physician recommendations do not necessarily result in colorectal screening (18,19), screening programs should be structured to enhance the effect of physician advice. Reliance on colonoscopy may not support adequate screening at the population level (4); 18.6% of our sample received physician advice for FOBT, whereas 51.3% received advice for colonoscopy. Population-based screening programs need to promote a more balanced approach (ie, multiple options) to screening recommendations. A balanced approach is important because limited access to gastroenterologists is linked with a high incidence of late-stage colorectal cancer in rural areas (20). Data showing a concentration of vulnerable populations and low screening rates in 3 rural public health regions of the state (10) underscore the importance of promoting a more balanced approach. Evidence that self-reported data overestimate screening rates (21) suggests that our data may underestimate the magnitude of the problem in rural public health regions. Based on policy initiatives recommending third-party reimbursement for all evidence-based screening methods (3) and state data demonstrating low screening rates in rural areas that have limited access to endoscopic screening, the Arkansas Colorectal Cancer Prevention, Early Detection, and Treatment Act of 2009 included language authorizing reimbursement for all evidence-based screening methods to make FOBT and endoscopic procedures equally available.

That black participants were less likely than white participants to report receiving physician advice may reflect physician expectations about patient interest or ability to pay for screening (22,23). Our study showed that a larger proportion of blacks than whites reported that physicians do not spend enough time during visits and do not involve them in health care decisions. Time spent with a physician and adequate understanding of physician recommendations predict screening (14,15); differences in time spent and involvement in decision making may contribute to disparities in screening. The Arkansas Colorectal Cancer Prevention, Early Detection, and Treatment Act of 2009 included language that called for activities to improve professional skills for enhancing the detection and control of colorectal cancer. Our study shows a need to promote knowledge and attitudes among health professionals that will help incorporate screening recommendations as a component of routine health care visits. Professional education is needed to increase awareness of state policies and resources for no-cost or minimal-cost screening for eligible participants, guidelines for multiple screening methods, and the importance of involving patients in health care decisions. Reimbursement to primary care providers for a health care visit that addresses screening recommendations may help support this recommendation.

This study has several limitations. Results and recommendations are based on self-reported data and are constrained by the limitations inherent in population-based surveys. Although the collection and application of state-specific data have been integral components of a collaborative process to establish population-based screening, demonstration of the effect of legislation and policies on colorectal cancer screening, incidence, or death rates does not fall within the scope of this study, and we cannot claim or measure any cause or effect. Recommendations for population-based screening in Arkansas have potential relevance for populations that have similar demographic profiles, but study results and policy outcomes may not generalize to other settings.

Our survey informed efforts to decrease disparities in colorectal cancer screening in Arkansas. State-specific data were used in conjunction with evidence-based guidelines to engage local stakeholders in a novel initiative to promote colorectal cancer screening. Grass roots, health policy, and legislative stakeholders collaborated to establish legislation intended to provide financial incentives for health care professionals to reduce out-of-pocket expenses for patients and promote a more balanced approach to screening. This process provides a heuristic model for others who wish to implement a population-based screening program that addresses colorectal cancer disparities.
